# Insulin use, hormone receptor status and hematopoietic cytokines׳ circulation in women with diabetes mellitus and breast cancer

**DOI:** 10.1016/j.dib.2017.02.037

**Published:** 2017-02-21

**Authors:** Zachary A.P. Wintrob, Jeffrey P. Hammel, George K. Nimako, Dan P. Gaile, Alan Forrest, Alice C. Ceacareanu

**Affiliations:** a State University of New York at Buffalo, Dept. of Pharmacy Practice, NYS Center of Excellence in Bioinformatics and Life Sciences, 701 Ellicott Street, Buffalo, NY 14203, USA; b Cleveland Clinic, Dept. of Biostatistics and Epidemiology, 9500 Euclid Ave., Cleveland, OH 44195, USA; c State University of New York at Buffalo, Dept. of Biostatistics, 718 Kimball Tower, Buffalo, NY 14214, USA; d The UNC Eshelman School of Pharmacy, Division of Pharmacotherapy and Experimental Therapeutics, Campus Box 7569, Chapel Hill, NC 27599, USA; e Roswell Park Cancer Institute, Dept. of Pharmacy Services, Elm & Carlton Streets, Buffalo, NY 14263, USA

**Keywords:** Hematopoiesis, Hematopoietic cytokines, G-CSF, GM-CSF, IL-7, Insulin, Breast cancer, Diabetes, Cancer outcomes, Cancer prognosis, Estrogen receptor, Progesterone receptor, ER, PR

## Abstract

Granulocyte colony-stimulating factor (G-CSF) and granulocyte macrophage colony-stimulating factor (GM-CSF) are cytokines of particular interest in oncology from the perspective of neutropenia management (Mehta et al., 2015 [1]) and also as indirect activators of tumor-associated macrophages and modifiers of tumor microenvironment. Associated with poor breast cancer survival and unfavorable hormone receptor status (Wintrob et al., 2017 [2]), insulin may also influence hematopoiesis, thus interfering with colony stimulating factor production. Although G-CSF has been linked to exacerbating insulin resistance (Ordelheide et al., 2016 [3]), thus far no study linked insulin treatment and hematopoietic cytokines production. Additionally, IL-7 is the primary driver of T and B cell differentiation, maturation, and response (Corfe and Paige, 2012 [4]) and its elevated levels have been associated with poor prognosis in breast cancer.

The data presented here is among the first to show a relationship between pre-existing use of injectable insulin in women diagnosed with breast cancer and type 2 diabetes mellitus, hematopoietic cytokine profiles at time of breast cancer diagnosis, and subsequent cancer outcomes. A Pearson correlation analysis evaluating the relationship between G-CSF, GM-CSF, and IL-7 stratified by insulin use, controls, as well as by estrogen and progesterone receptor status is also provided.

**Specifications Table**

**Table:** 

Subject area	Clinical and Translational Research
More specific subject area	Biomarker Research, Cancer Epidemiology
Type of data	Tables
How data was acquired	Tumor registry query was followed by vital status ascertainment, and medical records review
	Luminex®-based quantitation of hematopoietic cytokines (granulocyte colony stimulating factor, granulocyte macrophage colony stimulating factor, and interleukin- 7) from plasma samples was conducted.
	A Luminex®200^TM^ instrument with Xponent 3.1 software was used to acquire all data
Data format	Analyzed
Experimental factors	A total of 3 hematopoietic cytokines were determined from the corresponding plasma samples collected at the time of breast cancer diagnosis
Experimental features	The dataset included 97 adult females with diabetes mellitus and newly diagnosed breast cancer (cases) and 194 matched controls (breast cancer only). Clinical and treatment history were evaluated in relationship with cancer outcomes and hematopoietic cytokines profiles. A cytokine correlation analysis was also performed.
Data source location	United States, Buffalo, NY - 42° 53׳ 50.3592"N; 78° 52׳ 2.658"W
Data accessibility	The data is with this article

**Value of the data**

• This dataset represents the observed relationship between injectable insulin use, hormone receptor status, circulating hematopoietic cytokines at breast cancer diagnosis, and outcomes
• Reported data has the potential to guide future studies evaluating insulin-regulated hematopoiesis modulation in breast cancer as well as studies investigating the mechanism responsible to hormone receptor phenotype
• Our observations can assist further research clarifying the role of insulin in hematopoietic differentiation and biomarker signaling related to hormone receptor status
• This evidence builds on the existing cross-talk exploring the interaction between hematopoiesis and cancer

## 1 Data

Reported data represents the observed association between use of injectable insulin preceding breast cancer and the hematopoietic cytokine profiles at the time of cancer diagnosis in women with diabetes mellitus (Table 1). Data in Table 2 includes the observed correlations between hematopoietic cytokine stratified by type 2 diabetes mellitus pharmacotherapy and controls. Interferon α2 and γ correlation with each of the studied hematopoietic cytokine is presented in Table 2, however, the details regarding these biomarkers’ determination from plasma, association with cancer outcomes and use of injectable insulin is reported in a distinct dataset [6]. Table 3 provides the observed correlations stratified by hormone receptor status.

## 2 Experimental design, materials and methods

Evaluation of hematopoietic cytokine profile association with injectable insulin use and BC outcomes was carried out under two protocols approved by both Roswell Park Cancer Institute (EDR154409 and NHR009010) and the State University of New York at Buffalo (PHP0840409E). Demographic and clinical patient information was linked with cancer outcomes and hematopoietic cytokine profiles of corresponding plasma specimen harvested at BC diagnosis and banked in the Roswell Park Cancer Institute Data Bank and Bio-Repository.

### 2.1 Study population

All incident breast cancer cases diagnosed at Roswell Park Cancer Institute (01/01/2003-12/31/2009) were considered for inclusion (n=2194). Medical and pharmacotherapy history were used to determine the baseline presence of diabetes.

### 2.2 Inclusion and exclusion criteria

All adult women with pre-existing diabetes at breast cancer diagnosis having available banked treatment-naïve plasma specimens (blood collected prior to initiation of any cancer-related therapy - surgery, radiation or pharmacotherapy) in the Institute׳s Data Bank and Bio-Repository were included.

Subjects were excluded if they had prior cancer history or unclear date of diagnosis, incomplete clinical records, type 1 or unclear diabetes status. For a specific breakdown of excluded subjects, please see the original research article by Wintrob et al. [2].

A total of 97 female subjects with breast cancer and baseline diabetes mellitus were eligible for inclusion in this analysis.

### 2.3 Control-matching approach

Each of the 97 adult female subjects with breast cancer and diabetes mellitus (defined as “cases”) was matched with two other female subjects diagnosed with breast cancer, but without baseline diabetes mellitus (defined as “controls”). The following matching criteria were used: age at diagnosis, body mass index category, ethnicity, menopausal status and tumor stage (as per the American Joint Committee on Cancer). Some matching limitations applied [2].

### 2.4 Demographic and clinical data collection

Clinical and treatment history was documented as previously described [1]. Vital status was obtained from the Institute׳s Tumor Registry, a database updated biannually with data obtained from the National Comprehensive Cancer Networks’ Oncology Outcomes Database. Outcomes of interest were breast cancer recurrence and/or death.

### 2.5 Plasma specimen storage and retrieval

All the plasma specimens retrieved from long-term storage were individually aliquoted in color coded vials labeled with unique, subject specific barcodes. Overall duration of freezing time was accounted for all matched controls ensuring that the case and matched control specimens had similar overall storage conditions. Only two instances of freeze-thaw were allowed between biobank retrieval and biomarker analyses: aliquoting procedure step and actual assay.

### 2.6 Luminex® assays

A total of 3 biomarkers (granulocyte colony stimulating factor, granulocyte macrophage colony stimulating factor, and interleukin- 7) were quantified according to the manufacturer protocol. The HCYTOMAG-60K Luminex® biomarker panel (Millipore Corporation, Billerica, MA) was utilized in this study. Interferon α2 and γ determinations were done according to the manufacturer protocol as reported in our dataset focusing on Th1/Th2 cytokines׳ determinations [6].

### 2.7 Biomarker-pharmacotherapy association analysis

Biomarker cut-point optimization was performed for each analyzed biomarker. Biomarker levels constituted the continuous independent variable that was subdivided into two groups that optimized the log rank test among all possible cut-point selections yielding a minimum of 10 patients in any resulting group. Quartiles were also constructed. The resultant biomarker categories were then tested for association with type 2 diabetes mellitus therapy and controls by Fisher׳s exact test. The continuous biomarker levels were also tested for association with diabetes therapy and controls across groups by the Kruskall-Wallis test and pairwise by the Wilcoxon rank sum. Multivariate adjustments were performed accounting for age, tumor stage, body mass index, estrogen receptor status, and cumulative comorbidity. The biomarker analysis was performed using R Version 2.15.3. Please see the original article for an illustration of the analysis workflow [2].

Correlations between biomarkers stratified by type 2 diabetes mellitus pharmacotherapy, controls, and hormone receptor status were assessed by the Pearson method. Correlation models were constructed both with and without adjustment for age, body mass index, and the combined comorbidity index. The correlation stratification by hormone receptor status excluded 3 subjects that were estrogen receptor negative but progesterone receptor positive due to insufficient numbers to compute confidence intervals and correlation significance. Correlation analyses were performed using SAS Version 9.4.

## Funding sources

This research was funded by the following grant awards: Wadsworth Foundation Peter Rowley Breast Cancer Grant awarded to A.C.C. (UB Grant number 55705, Contract CO26588).

## Figures and Tables

**Table 1:** Hematopoietic cytokine associations with insulin use.

Biomarker	Biomarker	Concentration	Control	No Insulin	Any Insulin	Unadjusted p-value (MVP)			
						p^1^	p^2^	p^3^	Global
	Grouping								
									Test
G-CSF (pg/ml)	Median	–	30.82	36.99	45.68	0.044	0.060	0.400	0.035
	(25th–75th)		(20.84–47.23)	(27.34–61.00)	(27.89–75.49)	(0.870)	(0.640)	(0.450)	(0.770)
	Quartiles	1.60 to 21.73	55 (28.4%)	15 (19.7%)	3 (15.0%)	0.160	0.120	0.780	0.130
		21.84 to 33.05	52 (26.8%)	17 (22.4%)	4 (20.0%)				
		33.11 to 54.17	48 (24.7%)	20 (26.3%)	4 (20.0%)				
		54.29 to 2182.70	39 (20.1%)	24 (31.6%)	9 (45.0%)				
	OS-Based	1.60 to 12.99	22 (11.3%)	5 (6.6%)	1 (5.0%)	0.250	0.700	1.000	0.500
	Optimization	**13.06 to 2182.70**	172 (88.7%)	71 (93.4%)	19 (95.0%)	(0.590)	(0.910)	(0.990)	(0.830)
	DFS-Based	1.60 to 12.99	22 (11.3%)	5 (6.6%)	1 (5.0%)	0.250	0.700	1.000	0.500
	Optimization	**13.06 to 2182.70**⁎	172 (88.7%)	71 (93.4%)	19 (95.0%)	(0.590)	(0.910)	(0.990)	(0.830)
									
GM-CSF (pg/ml)	Median	–	4.95	5.92	5.06	0.210	0.630	0.690	0.430
	(25th–75th)		(3.48–9.08)	(3.51–9.45)	(3.41–13.06)	(0.130)	(0.460)	(0.760)	(0.250)
	Quartiles	0.64 to 3.46	49 (25.3%)	19 (25.0%)	5 (25.0%)	0.190	0.760	0.390	0.370
		3.52 to 5.29	54 (27.8%)	13 (17.1%)	5 (25.0%)				
		5.32 to 9.50	44 (22.7%)	25 (32.9%)	3 (15.0%)				
		9.64 to 1196.39	47 (24.2%)	19 (25.0%)	7 (35.0%)				
	OS-Based	0.64 to 2.10	20 (10.3%)	4 (5.3%)	2 (10.0%)	0.200	1.000	0.600	0.430
	Optimization	**2.20 to 1196.39**	174 (89.7%)	72 (94.7%)	18 (90.0%)	(0.220)	(1.000)	(0.260)	(0.430)
	DFS-Based	0.64 to 3.00	35 (18.0%)	12 (15.8%)	2 (10.0%)	0.660	0.540	0.730	0.680
	Optimization	**3.00 to 1196.39**	159 (82.0%)	64 (84.2%)	18 (90.0%)	(0.740)	(0.240)	(0.650)	(0.620)
									
IL-7 (pg/ml)	Median	–	0.58	0.82	1.00	0.027	0.070	0.540	0.028
	(25th–75th)		(0.36–1.76)	(0.44–2.34)	(0.44–3.72)	(0.160)	(0.045)	(0.190)	(0.060)
	Quartiles	0.19 to 0.38	52 (26.8%)	19 (25.0%)	5 (25.0%)	0.340	0.260	0.820	0.380
		0.45 to 0.58	59 (30.4%)	16 (21.1%)	3 (15.0%)				
		0.66 to 1.99	41 (21.1%)	19 (25.0%)	4 (20.0%)				
		2.05 to 70	42 (21.6%)	22 (28.9%)	8 (40.0%)				
	OS-Based	**0.19 to 0.96**	127 (65.5%)	40 (52.6%)	10 (50.0%)	0.052	0.180	0.830	0.090
	Optimization	0.98 to 70	67 (34.5%)	36 (47.4%)	10 (50.0%)	(0.200)	(0.640)	(0.850)	(0.400)
	DFS-Based	**0.19 to 0.96**	127 (65.5%)	40 (52.6%)	10 (50.0%)	0.052	0.180	0.830	0.090
	Optimization	0.98 to 70	67 (34.5%)	36 (47.4%)	10 (50.0%)	(0.200)	(0.640)	(0.850)	(0.400)

⁎ Overall survival (OS)- and disease-free survival (DFS)-optimized biomarker ranges associated with poorer outcomes are represented in bold. p^1^=pairwise comparison of controls with the no insulin group, p^2^= pairwise comparison of controls with the any insulin group, and p^3^=pairwise comparison of the no insulin and any insulin groups. Global Test=significance test across all groups. MVP= p-value of the multivariate adjusted analysis. Granulocyte colony stimulating factor (G-CSF), granulocyte macrophage colony stimulating factor (GM-CSF), and interleukin- 7 (IL-7).

**Table 2:** Hematopoietic cytokine correlations by insulin use.

			Unadjusted Correlation			Adjusted Correlation		
Compared Biomarkers		Group	Pearson Correlation	95% Confidence Interval	p-value	Pearson Correlation	95% Confidence Interval	p-value
G-CSF	GM-CSF	All Subjects (n=291)	**0.850**	**0.814 to 0.879**	**<0.001**	**0.850**	**0.814 to 0.880**	**<0.001**
		Controls (n=194)	**0.945**	**0.928 to 0.958**	**<0.001**	**0.945**	**0.927 to 0.958**	**<0.001**
		No Insulin (n=77)	**0.720**	**0.592 to 0.813**	**<0.001**	**0.739**	**0.614 to 0.826**	**<0.001**
		Any Insulin (n=20)	**0.484**	**0.053 to 0.763**	**0.026**	**0.609**	**0.182 to 0.843**	**0.006**
								
G-CSF	IL-7	All Subjects (n=291)	**0.210**	**0.097 to 0.317**	**<0.001**	**0.214**	**0.101 to 0.322**	**<0.001**
		Controls (n=194)	**0.193**	**0.054 to 0.325**	**0.007**	**0.200**	**0.060 to 0.332**	**0.005**
		No Insulin (n=77)	**0.788**	**0.685 to 0.860**	**<0.001**	**0.796**	**0.693 to 0.867**	**<0.001**
		Any Insulin (n=20)	0.255	−0.212 to 0.627	0.270	0.360	−0.146 to 0.716	0.147
								
G-CSF	IFN-α2	All Subjects (n=291)	**0.860**	**0.826 to 0.887**	**<0.001**	**0.861**	**0.828 to 0.888**	**<0.001**
		Controls (n=194)	**0.908**	**0.879 to 0.930**	**<0.001**	**0.907**	**0.878 to 0.929**	**<0.001**
		No Insulin (n=77)	**0.683**	**0.542 to 0.787**	**<0.001**	**0.707**	**0.571 to 0.805**	**<0.001**
		Any Insulin (n=20)	**0.556**	**0.150 to 0.801**	**0.008**	**0.655**	**0.254 to 0.864**	**0.003**
								
G-CSF	IFN-γ	All Subjects (n=291)	**0.461**	**0.365 to 0.547**	**<0.001**	**0.462**	**0.366 to 0.548**	**<0.001**
		Controls (n=194)	**0.505**	**0.392 to 0.603**	**<0.001**	**0.505**	**0.392 to 0.604**	**<0.001**
		No Insulin (n=77)	**0.467**	**0.271 to 0.625**	**<0.001**	**0.499**	**0.305 to 0.653**	**<0.001**
		Any Insulin (n=20)	0.421	−0.026 to 0.728	0.058	**0.514**	**0.045 to 0.798**	**0.029**
								
GM-CSF	IL-7	All Subjects (n=291)	**0.426**	**0.327 to 0.515**	**<0.001**	**0.429**	**0.330 to 0.519**	**<0.001**
		Controls (n=194)	**0.197**	**0.058 to 0.329**	**0.006**	**0.203**	**0.063 to 0.335**	**0.005**
		No Insulin (n=77)	**0.872**	**0.806 to 0.917**	**<0.001**	**0.871**	**0.802 to 0.917**	**<0.001**
		Any Insulin (n=20)	**0.859**	**0.673 to 0.943**	**<0.001**	**0.834**	**0.590 to 0.938**	**<0.001**
								
GM-CSF	IFN-α2	All Subjects (n=291)	**0.953**	**0.941 to 0.962**	**<0.001**	**0.953**	**0.941 to 0.962**	**<0.001**
		Controls (n=194)	**0.966**	**0.956 to 0.975**	**<0.001**	**0.967**	**0.956 to 0.975**	**<0.001**
		No Insulin (n=77)	**0.953**	**0.928 to 0.970**	**<0.001**	**0.953**	**0.926 to 0.970**	**<0.001**
		Any Insulin (n=20)	**0.990**	**0.973 to 0.996**	**<0.001**	**0.990**	**0.972 to 0.997**	**<0.001**
								
GM-CSF	IFN-γ	All Subjects (n=291)	**0.611**	**0.534 to 0.678**	**<0.001**	**0.612**	**0.534 to 0.679**	**<0.001**
		Controls (n=194)	**0.542**	**0.434 to 0.634**	**<0.001**	**0.543**	**0.434 to 0.636**	**<0.001**
		No Insulin (n=77)	**0.767**	**0.656 to 0.846**	**<0.001**	**0.765**	**0.650 to 0.846**	**<0.001**
		Any Insulin (n=20)	**0.780**	**0.515 to 0.909**	**<0.001**	**0.860**	**0.647 to 0.949**	**<0.001**
								
IL-7	IFN-α2	All Subjects (n=291)	**0.371**	**0.267 to 0.466**	**<0.001**	**0.370**	**0.266 to 0.466**	**<0.001**
		Controls (n=194)	**0.207**	**0.068 to 0.338**	**0.004**	**0.212**	**0.072 to 0.344**	**0.003**
		No Insulin (n=77)	**0.801**	**0.703 to 0.869**	**<0.001**	**0.801**	**0.700 to 0.870**	**<0.001**
		Any Insulin (n=20)	**0.800**	**0.554 to 0.918**	**<0.001**	**0.769**	**0.457 to 0.912**	**<0.001**
								
IL-7	IFN-γ	All Subjects (n=291)	**0.316**	**0.208 to 0.416**	**<0.001**	**0.321**	**0.213 to 0.421**	**<0.001**
		Controls (n=194)	**0.181**	**0.041 to 0.313**	**0.011**	**0.187**	**0.046 to 0.321**	**0.009**
		No Insulin (n=77)	**0.589**	**0.421 to 0.719**	**<0.001**	**0.589**	**0.417 to 0.721**	**<0.001**
		Any Insulin (n=20)	**0.555**	**0.149 to 0.801**	**0.008**	**0.720**	**0.365 to 0.892**	**<0.001**

Significant correlations are displayed in bolded text. The differences that are only significant in either adjusted or unadjusted correlations are further denoted by an outline. Granulocyte colony stimulating factor (G-CSF), granulocyte macrophage colony stimulating factor (GM-CSF), interleukin- 7 (IL-7), interferon α 2 (IFN-α2), and interferon γ (IFN-γ).

**Table 3:** Hematopoietic cytokine correlations by hormone receptor status.

			Unadjusted Correlation			Adjusted Correlation		
Compared Biomarkers		Group	Pearson Correlation	95% Confidence Interval	p-value	Pearson Correlation	95% Confidence Interval	p-value
G-CSF	GM-CSF	ER+/PR+ (n=179)	**0.918**	**0.890 to 0.938**	**<0.001**	**0.918**	**0.891 to 0.938**	**<0.001**
		ER+/PR- (n=36)	0.059	−0.276 to 0.379	0.736	0.002	−0.342 to 0.345	0.992
		ER-/PR- (n=57)	**0.464**	**0.228 to 0.644**	**<0.001**	**0.489**	**0.251 to 0.667**	**<0.001**
		Not Tested (n=16)	**0.799**	**0.481 to 0.923**	**<0.001**	**0.866**	**0.578 to 0.956**	**<0.001**
								
G-CSF	IL-7	ER+/PR+ (n=179)	**0.158**	**0.011 to 0.297**	**0.035**	**0.167**	**0.019 to 0.307**	**0.027**
		ER+/PR- (n=36)	0.165	−0.175 to 0.466	0.339	−0.051	−0.386 to 0.299	0.782
		ER-/PR- (n=57)	0.248	−0.015 to 0.476	0.062	**0.278**	**0.008 to 0.506**	**0.042**
		Not Tested (n=16)	**0.878**	**0.661 to 0.955**	**<0.001**	**0.914**	**0.712 to 0.972**	**<0.001**
								
G-CSF	IFN-α2	ER+/PR+ (n=179)	**0.895**	**0.860 to 0.920**	**<0.001**	**0.895**	**0.860 to 0.920**	**<0.001**
		ER+/PR- (n=36)	0.149	−0.191 to 0.454	0.388	0.077	−0.275 to 0.408	0.673
		ER-/PR- (n=57)	**0.465**	**0.228 to 0.644**	**<0.001**	**0.494**	**0.256 to 0.670**	**<0.001**
		Not Tested (n=16)	**0.79**	**0.463 to 0.920**	**<0.001**	**0.86**	**0.563 to 0.954**	**<0.001**
								
G-CSF	IFN-γ	ER+/PR+ (n=179)	**0.494**	**0.373 to 0.597**	**<0.001**	**0.497**	**0.376 to 0.600**	**<0.001**
		ER+/PR- (n=36)	**0.484**	**0.178 to 0.697**	**0.002**	**0.455**	**0.125 to 0.687**	**0.007**
		ER-/PR- (n=57)	0.203	−0.063 to 0.439	0.131	0.205	−0.068 to 0.447	0.137
		Not Tested (n=16)	**0.861**	**0.620 to 0.948**	**<0.001**	**0.908**	**0.695 to 0.970**	**<0.001**
								
GM-CSF	IL-7	ER+/PR+ (n=179)	**0.219**	**0.074 to 0.353**	**0.003**	**0.224**	**0.078 to 0.360**	**0.003**
		ER+/PR- (n=36)	−0.092	−0.407 to 0.245	0.594	0.576	−0.452 to 0.224	0.470
		ER-/PR- (n=57)	**0.607**	**0.407 to 0.747**	**<0.001**	**0.936**	**0.394 to 0.747**	**<0.001**
		Not Tested (n=16)	**0.975**	**0.922 to 0.991**	**<0.001**	**0.998**	**0.945 to 0.995**	**<0.001**
								
GM-CSF	IFN-α2	ER+/PR+ (n=179)	**0.961**	**0.948 to 0.971**	**<0.001**	**0.961**	**0.948 to 0.971**	**<0.001**
		ER+/PR- (n=36)	**0.643**	**0.391 to 0.799**	**<0.001**	**0.576**	**0.282 to 0.764**	**<0.001**
		ER-/PR- (n=57)	**0.937**	**0.893 to 0.962**	**<0.001**	**0.936**	**0.890 to 0.962**	**<0.001**
		Not Tested (n=16)	**0.998**	**0.995 to 0.999**	**<0.001**	**0.998**	**0.994 to 0.999**	**<0.001**
								
GM-CSF	IFN-γ	ER+/PR+ (n=179)	**0.613**	**0.511 to 0.696**	**<0.001**	**0.618**	**0.516 to 0.701**	**<0.001**
		ER+/PR- (n=36)	0.161	−0.179 to 0.463	0.350	0.245	−0.111 to 0.540	0.171
		ER-/PR- (n=57)	**0.412**	**0.166 to 0.605**	**0.001**	**0.409**	**0.155 to 0.607**	**0.002**
		Not Tested (n=16)	**0.979**	**0.934 to 0.992**	**<0.001**	**0.986**	**0.947 to 0.995**	**<0.001**
								
IL-7	IFN-α2	ER+/PR+ (n=179)	**0.227**	**0.083 to 0.361**	**0.002**	**0.234**	**0.088 to 0.369**	**0.002**
		ER+/PR- (n=36)	−0.039	−0.362 to 0.294	0.824	-0.056	−0.391 to 0.294	0.759
		ER-/PR- (n=57)	**0.655**	**0.471 to 0.780**	**<0.001**	**0.649**	**0.457 to 0.779**	**<0.001**
		Not Tested (n=16)	**0.966**	**0.896 to 0.988**	**<0.001**	**0.977**	**0.918 to 0.993**	**<0.001**
								
IL-7	IFN-γ	ER+/PR+ (n=179)	0.129	−0.019 to 0.270	0.086	0.136	−0.012 to 0.278	0.072
		ER+/PR- (n=36)	0.175	−0.165 to 0.474	0.309	−0.130	−0.452 to 0.225	0.472
		ER-/PR- (n=57)	0.246	−0.018 to 0.474	0.065	0.244	−0.028 to 0.479	0.075
		Not Tested (n=16)	**0.996**	**0.986 to 0.998**	**<0.001**	**0.997**	**0.989 to 0.999**	**<0.001**

Significant correlations are displayed in bolded text. The differences that are only significant in either adjusted or unadjusted correlations are further denoted by an outline. Granulocyte colony stimulating factor (G-CSF), granulocyte macrophage colony stimulating factor (GM-CSF), interleukin- 7 (IL-7), interferon α 2 (IFN-α2), interferon γ (IFN-γ), estrogen receptor (ER), progesterone receptor (PR), positive hormone receptor status (+), and negative hormone receptor status (-). Note that the hormone receptor category of ER-/PR+ was excluded due to an insufficient number of subject presenting that phenotype (n=3).
